# Mice Lacking NMDA Receptors in Parvalbumin Neurons Display Normal Depression-Related Behavior and Response to Antidepressant Action of NMDAR Antagonists

**DOI:** 10.1371/journal.pone.0083879

**Published:** 2014-01-16

**Authors:** Laura Pozzi, Iskra Pollak Dorocic, Xinming Wang, Marie Carlén, Konstantinos Meletis

**Affiliations:** Department of Neuroscience, Karolinska Institutet, Stockholm, Sweden; Sapienza University of Rome, Italy

## Abstract

The underlying circuit imbalance in major depression remains unknown and current therapies remain inadequate for a large group of patients. Discovery of the rapid antidepressant effects of ketamine - an NMDA receptor (NMDAR) antagonist – has linked the glutamatergic system to depression. Interestingly, dysfunction in the inhibitory GABAergic system has also been proposed to underlie depression and deficits linked to GABAergic neurons have been found with human imaging and in post-mortem material from depressed patients. Parvalbumin-expressing (PV) GABAergic interneurons regulate local circuit function through perisomatic inhibition and their activity is NMDAR-dependent, providing a possible link between NMDAR and the inhibitory system in the antidepressant effect of ketamine. We have therefore investigated the role of the NMDAR-dependent activity of PV interneurons for the development of depression-like behavior as well as for the response to rapid antidepressant effects of NMDAR antagonists. We used mutant mice lacking NMDA neurotransmission specifically in PV neurons (PV-Cre+/NR1f/f) and analyzed depression-like behavior and anhedonia. To study the acute and sustained effects of a single NMDAR antagonist administration, we established a behavioral paradigm of repeated exposure to forced swimming test (FST). We did not observe altered behavioral responses in the repeated FST or in a sucrose preference test in mutant mice. In addition, the behavioral response to administration of NMDAR antagonists was not significantly altered in mutant PV-Cre+/NR1f/f mice. Our results show that NMDA-dependent neurotransmission in PV neurons is not necessary to regulate depression-like behaviors, and in addition that NMDARs on PV neurons are not a direct target for the NMDAR-induced antidepressant effects of ketamine and MK801.

## Introduction

Drugs currently used for the treatment of major depression target monoaminergic neurotransmission, primarily serotonin and noradrenaline pathways, such as the selective serotonin and noradrenaline reuptake inhibitors. Current antidepressant treatments result in an inadequate therapeutic response due to the long delay of activity and failure of response in many patients [1]. There is therefore great clinical need for improved and rapid acting antidepressants.

Recent insights relevant for the development of faster acting antidepressants have come from the discovery that compounds targeting the glutamatergic system have acute antidepressant effects [2]. Interestingly, both preclinical animal models and recent clinical trials have reported efficacy of a single administration of the N-methyl-D-aspartate (NMDA) receptor antagonist ketamine on depressive behaviors, with effects that can last for several days [3]–[6]. In rodents, antidepressant-like effects after acute NMDA receptor (NMDAR) antagonist treatment have been observed in many models of depression, including inescapable stress, the forced swimming test, the tail suspension test, learned helplessness models of depression, and exposure to chronic mild stress procedures [3], [7]–[9]. This suggests that glutamate NMDAR antagonist-based treatments might represent an effective alternative to current therapies to treat depression [10], [11].

It is now well recognized that in addition to ketamine and MK801 [4], [9], [12], [13] various NMDAR antagonists such as amantadine and memantine can exhibit antidepressant activity in patients and in a range of preclinical screening procedures (reviewed in [14]). However, although their mechanism of action involves the inhibition of the NMDAR, the neuronal subtypes involved and the primary pharmacological target resulting in the antidepressant effects have not been established.

As a result of the significant clinical and preclinical observations described above, much effort is currently put into understanding the cellular and molecular mechanisms associated with antidepressant actions of NMDAR antagonists. Understanding the cellular targets and mechanisms by which NMDAR antagonist exert their antidepressant-like activity will facilitate our comprehension of depression and will help in developing improved therapeutic compounds.

The gamma-aminobutyric acid (GABA)-ergic inhibitory system constitutes a diverse class of neurons that play critical roles in regulating excitatory glutamatergic transmission and shape the global balance of activity in the brain. The GABAergic system has been proposed to be dysfunctional in mood disorders (reviewed in [15]), and deficiencies in the GABAergic system in patients with major depression have been demonstrated with imaging or in post-mortem material [16]–[20]. The behavioral relevance of the GABAergic system has also been demonstrated, both with pharmacological (reviewed in [21]) and genetic means [22], [23] as well as lately with optogenetic tools [24]. Of the inhibitory neurons, fast-spiking interneurons expressing the calcium binding protein parvalbumin (PV) have drawn particular interest, with several studies demonstrating their importance in fundamental cortical processes including generation of gamma oscillations [25], [26].

Gamma oscillations are tightly linked to cognitive functions [27] and perturbation of PV inhibition disrupts gamma oscillations and impairs cognitive functions [28]–[30]. It has been widely proposed that the GABAergic interneurons, and more specifically the PV interneurons, are a primary target of the non-competitive NMDAR antagonists [31]–[34]. This has been confirmed in studies where ablation of NMDAR specifically in PV interneurons in a rodent model results in markedly reduced sensitivity to the locomotor effects of MK801 [25].

In line with this, it has been proposed that fast-spiking PV interneurons play an important role in the antidepressant effects of ketamine [35].

Interestingly, GABAergic PV interneurons express NMDARs and receive a strong NMDA-dependent excitatory input from pyramidal cells [36]. NMDARs preferentially regulate the firing rate of GABAergic interneurons and pharmacological inhibition of NMDARs reduces their activity, resulting in a disinhibition of glutamate transmission through the increased firing rate of pyramidal neurons [32]. Thus, PV interneurons may regulate the activity of neural networks through NMDAR-dependent GABAergic disinhibition of local excitatory neurons. In summary, PV interneurons are perfectly positioned to regulate excitability and activity of local circuits and could underlie the antidepressant effects of NMDAR antagonists including ketamine.

To directly test the role of NMDAR function in PV neurons for depressive-like behaviors, we used genetically modified mice lacking the NR1 subunit specifically in PV neurons (i.e. PV-Cre+NR1f/f mice) [25]. Genetic deletion of the essential NR1 subunit in PV neurons in PV-Cre+ NR1f/f mice leads to a the functional loss of NMDAR currents, which has previously been demonstrated by whole cell recordings studies in hippocampal slices *in vitro*, showing that NMDAR-mediated synaptic transmission was abolished in PV interneurons [25].

In the present study the PV-Cre+NR1f/f mice were tested and in the reward based- sucrose preference test (SPT) and in a model of behavioral despair (i.e. forced swimming test, FST) to score the antidepressant-like effects [37] of the non-competitive glutamate NMDAR antagonists ketamine and MK801 using doses and routes of administration of ketamine and MK801 previously shown to produce behavioral effects in this test [3].

Specifically, we tested how the selective ablation of NMDAR neurotransmission selectively in PV cells affects a) the spontaneous responses to the FST and sucrose preference test and b) whether the acute (30 min) and sustained (24 h and 1 week) antidepressant-like effect of ketamine and MK801 in the FST depends on NMDARs on PV cells.

In summary, we found that loss of NMDAR selectively on PV neurons is not sufficient to induce changes in the FST behavior and in sucrose preference. In addition we could demonstrate that NMDAR antagonist-induced acute antidepressant-like effects are not dependent on NMDARs transmission in PV neurons.

## Materials and Methods

### Animals

All procedures and experiments were approved by the local animal ethics committees of Stockholm North and Karolinska Institutet in Sweden (approval N160/12). To generate mice lacking NMDAR specifically in PV interneurons (PVCre/NR1f/f mice) PV-Cre+ mice [38] were crossed to mice carrying ‘floxed’ NR1 alleles [39]. The study was performed in adult C57BL/6N, PV-Cre+/+NR1f/f and NR1f/f male mice of 13–16 weeks age. C57BL/6N mice were purchased from Charles River and habituated to the new environment for a minimum of 1 week before any experimental manipulation. PV-Cre+/NR1f/f and NR1f/f mice were bred at the Karolinska Institute, Sweden. Animals were housed in groups (3–5 mice/cage) using Makrolon type III cages, under standardized conditions with a 12-hour light-dark cycle (light 7:00am) and stable temperature (20±1°C) and humidity (40 to 50%) with access to food and water ad libitum.

### Genotyping of transgenic animals

DNA samples were prepared from mouse ear snips using NaOH extraction, and analyzed by PCR. PCR was performed using Quick-Load Taq 2× Master Mix (New England Biolabs). PCR conditions: denaturation at 94°C for 5 min followed by 30× 94°C for 30 s, 58°C for 30 s, 68°C for 45 s, 68°C for 5 min. Primers specific for Cre allele, internal control, floxed NR1 alleles were according to reference [25]. PCR products were analyzed by electrophoresis on 2% agarose with GelRed (Biotium).

### Behavioral experiments

All behavioral sessions were conducted between 14:00 and 16:00 p.m. The mice were transported to the testing room at least 1 h before any procedures to facilitate adaptation to the surroundings. All mice were habituated to the handler for 2–3 days prior the test day. At the test day the mice were weighed, marked and randomly assign to a drug treatment.

### Forced swimming test

The forced swimming test (FST) was performed as follow: mice were individually placed in a transparent acrylic cylindrical beaker (height: 60 cm, diameter: 14 cm) containing 1800 ml of clear water at 25±1° C for 6 min. Mice were not able to touch the bottom of the beaker with their tail. Water was changed between subjects. Passive floating was scored as immobility. A mouse was judged to be immobile when it remained floating passively in the water and to be swimming when it was actively making swimming movements that caused it to move around the cylinder. A decrease in immobility time indicates an antidepressant-like response. Immobility and swimming were measured the 4 last min of the test using automated software (Biobserve, Germany).

#### Experiment 1

On the experimental day, 12 wild type C57BL/6N male mice received an intraperitoneal (i.p.) injection of saline and subjected to the FST 30 min later. The same mice were retested in the FST 24 h, 48 h, 72 h, 96 h and 240 h later with no further treatment.

#### Experiment 2

On the experimental day 10 PV-Cre+/+NR1f/f and 10 NR1f/f male mice received an i.p. injection of saline and subjected to the FST 30 min later. The same mice were retested in the FST 24 h, 48 h, 72 h, 96 h and 240 h later with no further treatment.

#### Experiment 3

Four groups of C57BL/6N mice were injected i.p. with saline (n = 8), ketamine (n = 8), water (n = 10) or MK801 (n = 10) and subjected to the FST 30 min later. The same mice were retested in the FST 24 h and 1 week later with no further treatment.

#### Experiment 4

Two groups of PV-Cre+/NR1f/f and NR1f/f mice were used. The first group of mice received ketamine i.p. (PV-Cre+/NR1f/f n = 9 and NR1f/f n = 6) and subjected to the FST 30 min later. The second group received MK801 i.p. (PV-Cre+/NR1f/f n = 14 and NR1f/f n = 13) and subjected to the FST 30 min later. The same groups of mice were retested 24 h later with no further treatment.

After each session mice were taken out of the water and dried with a soft towel before being returned to their home cages. Each session was videotaped using a video camera (Sony 1/3″ CCD High Resolution 25 frame/sec) positioned in front of the cylinders (approximately 80 cm far). Data were analyzed in real time using the Forced Swim Test software (BIOBSERVE GmbH, DE).

### Sucrose preference test

Two groups of NR1f/f (n = 6) and PV-Cre+/+NR1f/f (n = 9) mice were single housed in clean cages and habituated overnight to the experimental room. A 24 h water drinking pre-test was carried out simultaneously in both groups of mice in a two-bottle paradigm.

Twenty four hours later mice were given a free choice between two bottles, one with 2% sucrose solution and another with tap water for 72 h. No previous food or water deprivation was applied before the test. Precautions concerning possible liquid spillage were applied. Bottles were filled in advance and kept overnight in the same room where the testing took place. This measure prevents the physical effect of liquid leakage resulting from growing temperature of air and pressure inside the bottles. To prevent the possible effects of a side-preference in drinking behavior, the position of the bottles in the cages was switched every 24 h during the test. The intake of water and 2% sucrose solution and total intake was estimated by weighing the bottles before and after access to liquids. The preference for sucrose was calculated as the percentage of the sucrose solution consumed out of the total amount of liquid drunk (sucrose solution intake/total intake ×100). Drinking bottles and tap water were the same such as those employed in the above-described water intake pre-test.

### Open field analysis

Mice were tested in an open field to examine whether ketamine or MK801, at the same dose which had effects in the FST (3 and 0.1 mg/kg i.p., respectively), alter locomotor activity. Locomotor activity (distance traveled expressed as cm) in a novel open field was measured in boxes with sets of 16 light beam arrays. During the experiment the hardware detected beams broken by the animal, with the software determining the location and activity of the animal. For the pharmacological treatment, C57BL/6N mice were first monitored in the open field for 60 min. After habituation mice received saline (10 ml/kg, n = 5), ketamine (3 mg/kg, n = 6) or MK801 (0.1 mg/kg, n = 5). All drugs were freshly prepared and administered i.p. After treatments mice were monitored for additionally 60 min, in the same boxes as before. Each point is the mean of 5 consecutive min. Six boxes (50×50×50 cm) were run simultaneously.

### Drugs

Ketamine (Ketaminol; Intervet) was dissolved in saline and injected at 3 mg/kg. MK801 (M-107; Sigma-Aldrich Sweden AB) was dissolved in ultra-pure distilled water and injected at 0.1 mg/kg. All drugs were injected i.p. in a volume of 10 ml/kg of body weight. Drugs were freshly prepared on each test day from a 1 mg/ml (MK801) and 50 mg/ml (ketamine) stock solutions. Doses, times and route of administration were selected based on the observations indicating that both drugs significantly reduced the immobility time in the same behavioral paradigm (FST) at different time points (3).

### Statistics

The data fulfilled the criteria for normal distribution. Therefore, to evaluate the effect of test compounds in the FST, behavioral parameters were analyzed by t-tests for the two genotypes or treatments and repeated ANOVA one way for the different days of tests. Only values of *p*<0.05 were considered statistically significant.

## Results

### Establishing a behavioral paradigm of repeated forced swimming test

**Figure 1:**
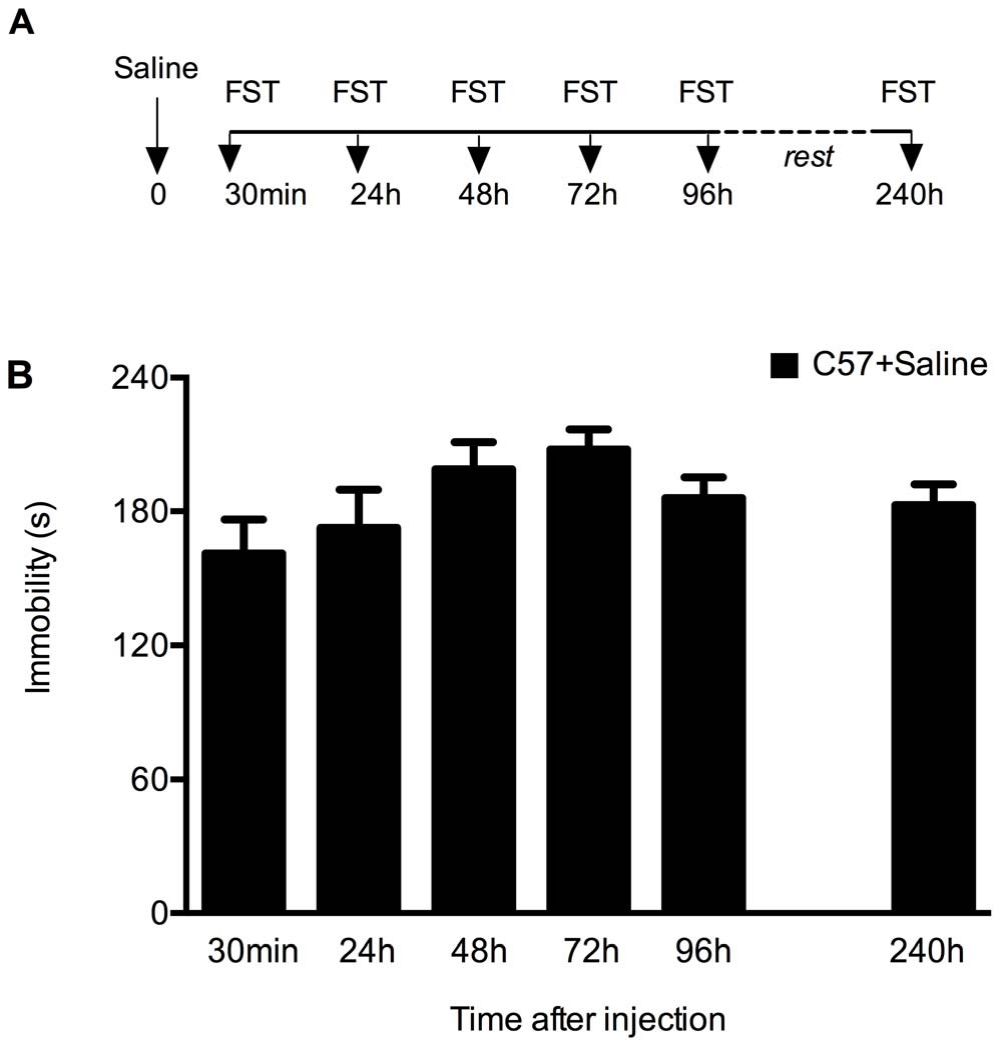
Mice display stable behavior in a repeated FST paradigm. (A) Outline of the repeated FST protocol performed over several days. C57BL/6N mice (n = 12) were injected at time 0 with saline (arrow) and every mouse was subjected to FST 30 min, 24 h, 48 h, 72 h and 96 h later. After 4 days of rest in their home cages mice were retested in the FST once again (i.e. 240 h after saline injection). (B) Results (sec/4 min) are presented as mean values ±SEM. Immobility time is stable after repeated FST: 161±15 (30 min); 173±17 (24 h); 199±12 (48 h); 208±9 (72 h); 186±9 (96 h) and 183±9 (240 h) (p>0.05, one way ANOVA).

We established a long-term repeated exposure to FST that allows for study of sustained effects of single administration of antidepressants over several days in the same animal (Fig. 1A). Twelve wild type C57BL/6N male mice received 10 ml/kg saline i.p 30 min before the first FST test. The mice were re-tested in the FST 24 h, 48 h, 72 h and 96 h after saline injection. After four days of rest, the same mice were retested in the FST 240 h ( = 10 days) after saline treatment. On each occasion the immobility time was measured in the last 4 min of the 6 min sessions (Fig. 1B). There was no significant change in the immobility time over the whole experimental period, demonstrating the stability of the behavioral response in this repeated FST exposure paradigm.

### Behavioral responses of PV-Cre+/NR1f/f mice in the repeated forced swim test

**Figure 2:**
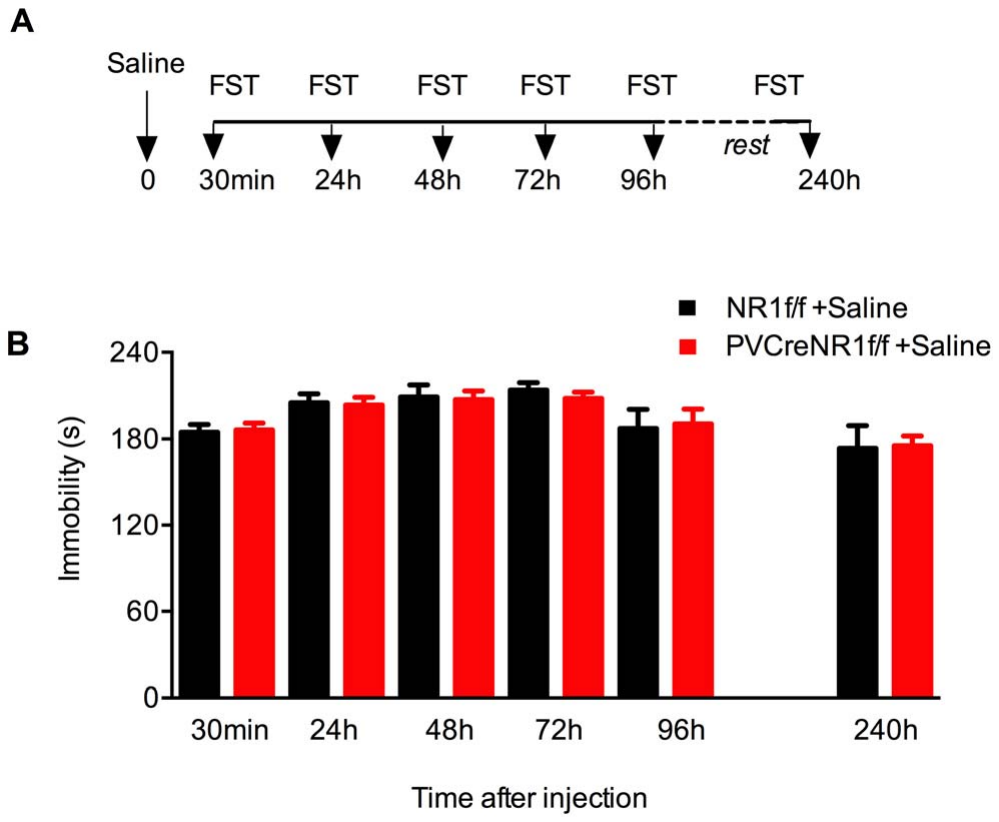
Loss of NMDAR in PV neurons does not affect behavior in the repeated FST paradigm. Immobility of mice lacking NMDAR specifically in PV interneurons (PV-Cre+/NR1f/f,*red bars*) and control mice (NR1f/f, *black bars*) during repeated FST. (A) Outline of the repeated FST protocol performed over several days. PV-Cre+/NR1f/f and NR1f/f mice were injected at time 0 with saline (arrow) and every mouse was subjected to FST 30 min, 24 h, 48 h, 72 h and 96 h later. After 4 days of rest in their home cages mice were retested in the FST once again (i.e. 240 h after saline injection). (B) Immobility time is stable after repeated FST in both genotypes. Results (sec/4 min) are presented as mean values ±SEM. Immobility time for NR1f/f mice (*black bars*, n = 10) is:185±5 (30 min), 205±6 (24 h), 209±8 (48 h), 214±5 (72 h), 187±13 (96 h) and 174±16 (240 h). Immobility time for PV-Cre+/NR1f/f mice (*red bars*, n = 10) is: 186±5 (30 min), 203±7 (24 h), 207±6 (48 h), 208±4 (72 h), 191±10 (96 h) and 175±7 (240 h).

To determine whether the lack of NMDA-dependent activity of PV interneurons results in a behavior that mimics antidepressant-like effects, we compared the behavior of 10 adult PV-Cre+/NR1f/f mutant mice and 10 control littermates (NR1f/f mice) in the repeated FST paradigm (Fig. 2). Both groups of mice received a single i.p. injection of 10 ml/kg saline and then tested in the FST 30 min, 24 h, 48 h, 72 h, 96 h and 240 h later as described above (Fig. 1A). We found that the immobility time was stable during the different days of exposure to FST for both PV-Cre+/NR1f/f mutant mice and NR1f/f mice (Fig. 2). Surprisingly, both genotypes displayed similar levels of immobility in the FST at all times point (*p*>0.05 one way ANOVA) (Fig. 2).

These data indicate that in contrast to the hypothesized function of NMDAR located in PV interneurons in the behavioral effect observed in the FST, animals that lack NMDA neurotransmission selectively in PV neurons do not to display antidepressant-like behavior as determined by immobility in the FST.

### Acute and sustained antidepressant effects of ketamine and MK801 in C57 mice

**Figure 3:**
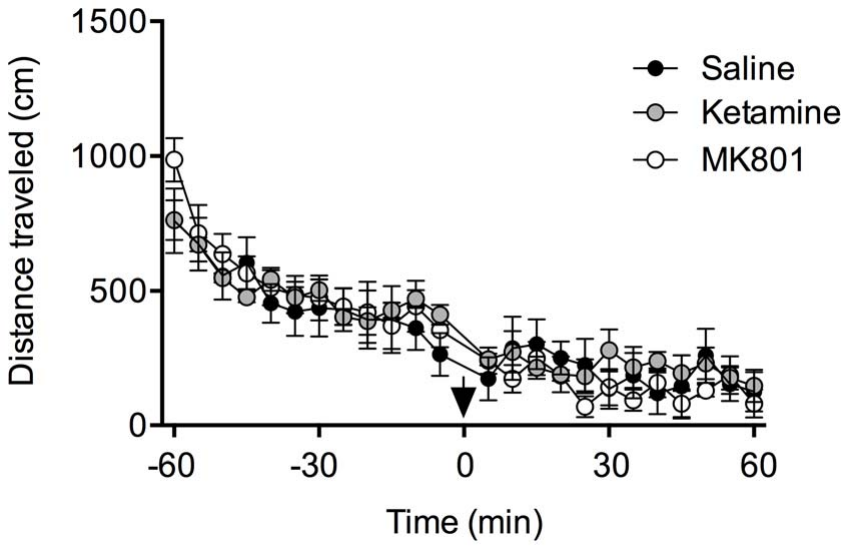
Ketamine and MK801 do not affect locomotor activity behavior in the open field. Time course for the locomotor activity of C57BL/6N mice in the 60 min before and 60 min after saline (*black circles*, n = 5), ketamine (*gray circles*, n = 6) and MK801 (*white circles*, n = 5) injection (arrow) (p>0.05, one way ANOVA).

**Figure 4:**
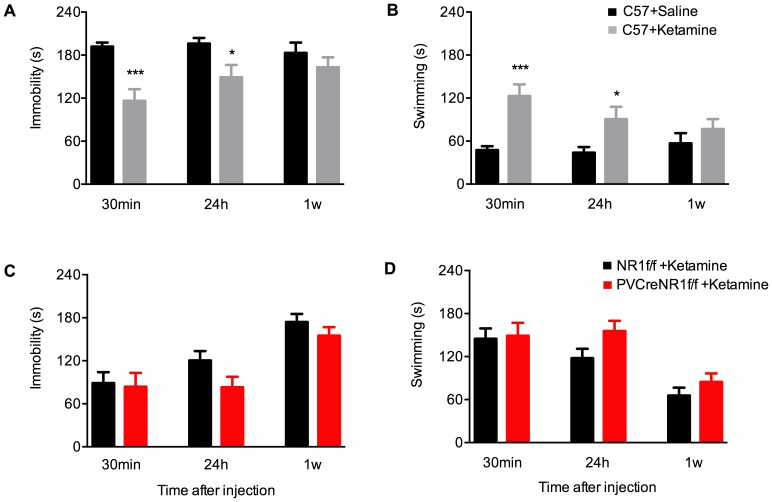
Ketamine-induced antidepressant-like behavioral effects in the FST. Ketamine-induced immobility and swimming time, respectively, in C57BL/6N mice (A,B) and in mice lacking NMDAR specifically in PV neurons (PV-Cre+/NR1f/f, *red bars*) compared with control (NR1f/f, *black bars*)(C,D). (A) Immobility of C57BL/6N mice in repeated FST 30 min, 24 h and 1 week after acute treatment with 3.0 mg/kg ketamine (*grey bars*, n = 8) or saline (*black bars*, n = 8). Results (sec/4 min) are presented as mean values ±SEM. Ketamine induces a rapid antidepressant response by significantly reducing immobility already 30 min after treatment. The antidepressant effect decreases over time. Ketamine-induced immobility time is: 192±5 saline and 116±16 ketamine (30 min) (p<0.001 Student's t-test); 196±8 saline and 149±17 ketamine (24 h) (p<0.05 Student's t-test); 183±14 saline and 163±14 ketamine (1 week) (p>0.05 Student's t-test). (B) Swimming scored in the experiment in (A). Ketamine-induced swimming time is: 47±5 saline and 123±16 ketamine (30 min) (p<0.001 Student's t-test); 44±8 saline and 91±17 ketamine (24 h) (p<0.05 Student's t-test); 57±14 saline and 77±14 ketamine (1 week) (p>0.05 Student's t-test). (C) Immobility of PV-Cre+/NR1f/f and NR1f/f mice in repeated FST after acute treatment with 3.0 mg/kg ketamine. Immobility time 30 min, 24 h and 1 week after ketamine is: 89±15, 121±13 and 174±11 (NR1f/f *black bars*, n = 6); 84±19, 83±14 and 155±12 (PV-Cre+/NR1f/f *red bars*, n = 9), respectively. (D) Swimming scored in the experiment in (C). Swimming time 30 min, 24 h and 1 week after ketamine is: 145±14, 118±13 and 66±11 (NR1f/f *black bars*, n = 6); 149±18, 156±14 and 85±12 (PV-Cre+/NR1f/*red bars*, n = 9), respectively. Both genotypes display similar behavioral response to the ketamine treatment. Results (sec/4 min) are presented as mean values ±SEM.

In order to examine the acute as well as long-term antidepressant effects of NMDAR antagonists ketamine and MK801, we first used naïve C57BL/6N male mice. To first evaluate a potential confounding influence of drug treatment on general locomotion, we assessed the effects of a single injection of ketamine (3 mg/kg) or MK801 (0.1 mg/kg) on spontaneous locomotors activity in the open field using the same drug concentration as for the FST (Fig. 3). Locomotion was scored during 60 min after drug administration. We found no significant effects of drug treatment in C57BL/6N mice. The lack of any effects of MK801 on locomotor activity in the open field test in NR1f/f and PV-Cre+/NR1f/f mice has been already reported [25]. We then used naïve C57BL/6N male mice that were given a single i.p. injection of ketamine (3 mg/kg), or its control solution saline, or MK801 (0.1 mg/kg), or its control solution water. All four animal groups were subjected to FST 30 min, 24 h and 1 week after injection. Both ketamine and MK801 significantly reduced immobility and increased the swimming time compared with respective control mice 30 min after injection, indicating their antidepressant-like activity. Specifically, acute treatment with ketamine reduced the immobility time by 40% at 30 min compared to control treatment with saline (Fig. 4A; *p*<0.001; Student's *t*-test). The same group of mice treated with ketamine displayed increased swimming at this time point by 156% over saline (Fig. 4B; *p*<0.001; Student's *t*-test).

**Figure 5:**
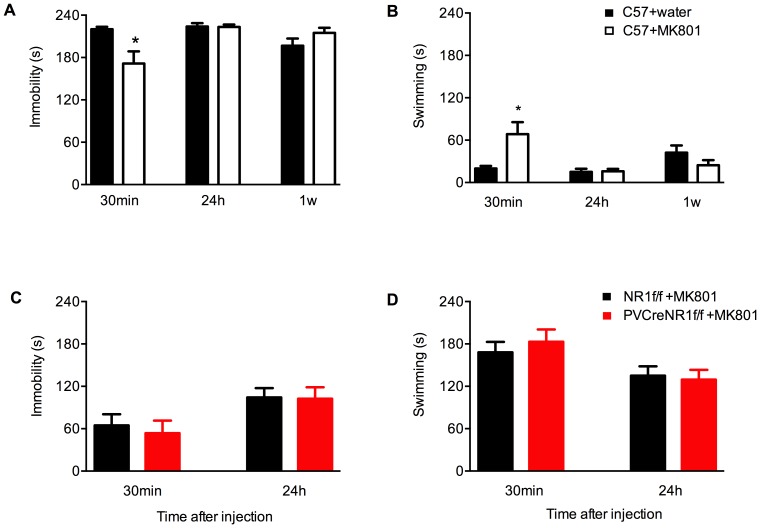
MK801-induced antidepressant-like behavioral effects in the FST. MK801-induced immobility and swimming time in C57BL/6N mice (A,B) and in mice lacking NMDAR specifically in PV neurons (PV-Cre+/NR1f/f, *red bars*) compared with control (NR1f/f, *black bars*) (C,D). (A) Immobility of C57BL/6N mice in repeated FST 30 min, 24 h and 1 week after acute treatment with 0.1 mg/kg MK801 (*white bars*, n = 10) or water (*black bars*, n = 10). MK801 induces rapid antidepressant-like responses to a lesser extent than ketamine 30 min after treatment. The antidepressant effect of MK801 is abolished 24 h and 1 week after treatment. MK801-induced immobility time is: 220±3 water and 171±17 MK801 (30 min) (p<0.05 Student's t-test); 224±5 water and 223±3 MK801 (24 h) (p>0.05 Student's t-test); 197±10 water and 215±7 MK801 (1 week) (p>0.05 Student's t-test). (B) Swimming scored in the experiment in (A). MK801-induced swimming time is: 20±3 water and 68±17 MK801 (30 min) (p<0.05 Student's t-test); 15±4 water and 16±3 MK801 (24 h) (p>0.05 Student's t-test); 42±10 water and 24±7 MK801 (1week) (p>0.05 Student's t-test). (C) Immobility of PV-Cre+/NR1f/f (*red bars*) and NR1f/f (*black bars*) mice in repeated FST 30 min and 24 h after acute treatment with 0.1 mg/kg MK801. Immobility time 30 min and 24 h after MK801 is: 65±16 and 104±13 (NR1f/f *black bars*, n = 13); 54±16 and 103±16 (PV-Cre+/NR1f/f *red bars*, n = 14), respectively. (D) Swimming scored in the experiment in (C). Swimming time 30 min and 24 h after MK801 is: 168±15 and 135±16 (NR1f/f *black bars*, n = 13); 183±15 and 130±14 (PV-Cre+/NR1f/f *red bars*, n = 14), respectively. All time points p>0,05 (Student's t-test). Both genotypes display similar behavioral response to the MK801 treatment. Results (sec/4 min) are presented as mean values ±SEM.

Treatment with a single dose of MK801 significantly reduced the immobility time 30 min after dosing, but to a lesser extent than treatment with ketamine. MK801-treated animals displayed 22% reduced immobility compared to water-treated control animals (Fig. 5A; *p*<0.05 Student's *t*-test). In accordance with this, acute treatment with MK801 resulted in increased swimming time (240%) 30 min after dosing compared to control treatment with water (Fig. 5B; *p*<0.05; Student's *t*-test).

In order to examine the time course of the behavioral antidepressant effects of a single dose of ketamine or MK801, the four groups of mice were retested in the FST paradigm 24 h and 1 week after drug treatment. The antidepressant effect of ketamine was still evident 24 h after treatment (24% less immobility than control mice) (Fig. 4A; *p*<0.05 Student's *t*-test). This effect was also reflected in the increased time spent swimming at this time point (106% over saline) (Fig. 4B: *p*<0.05 Student's *t*-test). However, 24 h after the administration of MK801, drug-treated and control animals displayed similar levels of immobility and swimming (Fig. 5A,B; *p*>0.05 Student's *t*-test).

One week after drug administration, the antidepressant effect of ketamine was no longer evident, with ketamine- and saline-treated mice displaying similar levels of immobility (Fig. 4A; *p*>0.05 Student's *t*-test) and swimming (Fig. 4B; *p*>0.05 Student's *t*-test). At this time point MK801-treated animals also displayed similar behavior as control animals, with long immobility times (Fig. 5A; *p*>0.05 Student's *t*-test) and little swimming (Fig. 5B; *p*>0.05 Student's *t*-test).

### Antidepressant responses to ketamine and MK801 in PV-Cre+/NR1f/f mice

As mice lacking NMDAR specifically in PV interneurons do not behave differently than their control littermates in the repeated exposure to FST as reported in Fig. 2, we next aimed to determine whether the acute antidepressant-like effects of NMDAR antagonists in FST were dependent on the presence of NMDAR in PV neurons (Figs. 4 and 5). We therefore treated PV-Cre+/NR1f/f and littermates NR1f/f control mice with ketamine (3 mg/kg) (Fig. 4C,D) or MK801 (0.1 mg/kg) (Fig. 5C,D) and tested their behavioral response in the FST 30 min and 24 h later.

Surprisingly, 30 min after ketamine injection we did not observe behavioral differences in the time spent floating (Fig. 4C) between PV-Cre+/NR1f/f and NR1f/f mice (*p*>0.05, Student's t-test). Twenty-four hours and 1 week after ketamine administration the immobility time in mutant PV-Cre+/NR1f/f mice tended to be lower compared with the immobility time in NR1f/f (Fig. 4C) yet this difference did not reach statistical significance (p>0.05, Student's t-test). No differences were found in the swimming time between the two groups of mice at 30 min 24 h and 1 week after ketamine administration (Fig. 4D; p>0.05, Student's t-test).

Paralleling the results found with ketamine administration, both genotypes responded similarly to treatment with MK801, with no differences found in the immobility at 30 min and 24 h (both *p*>0.05, Student's t-test)(Fig. 5C) or swimming at 30 min and 24 h (both *p*>0.05, Student's t-test (Fig. 5D).

### Behavioral responses of PV-Cre+/NR1f/f mice in the sucrose preference test

**Figure 6:**
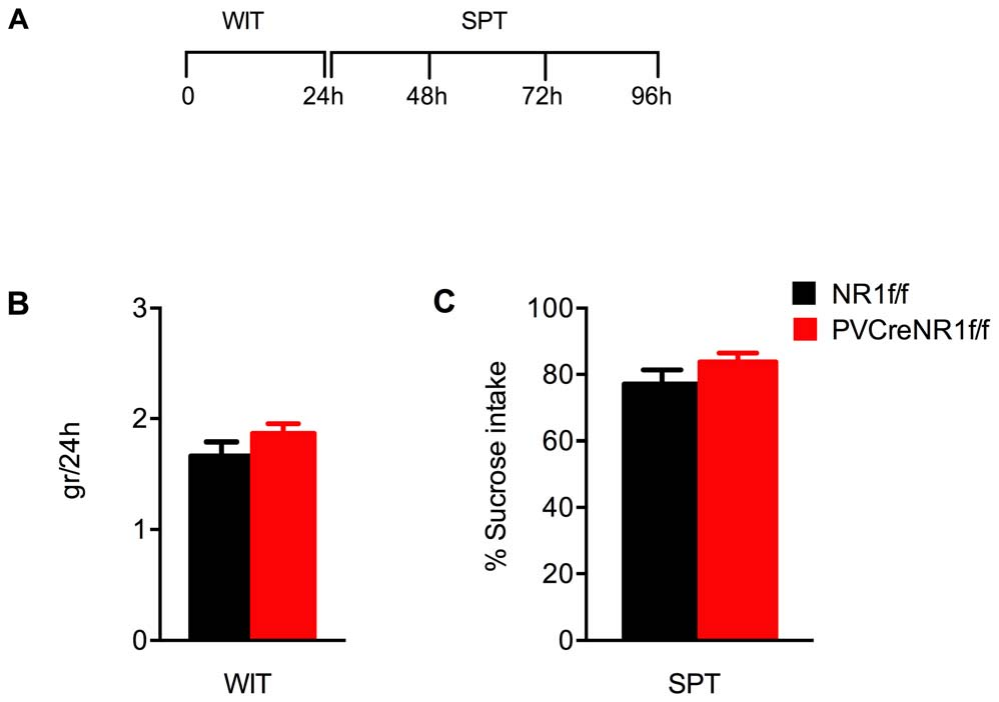
Sucrose preference test in animals lacking NMDAR in PV neurons. (A) Outline of the water intake and the sucrose preference test in PV-Cre+/NR1f/f and NR1f/f mice. (B) Water intake test (WIT) in mice lacking NMDAR specifically in PV neurons (PV-Cre+/NR1f/f, *red bars*, n = 9) compared with control mice (NR1f/f, *black bars*, n = 6). WIT was run for 24 h. Results (gr) are presented as mean values ±SEM (p>0,05;Student's t-test). (C) Sucrose preference test (SPT) in mice lacking NMDAR specifically in PV neurons (PV-Cre+/NR1f/f, *red bars*, n = 9) compared with control mice (NR1f/f, *black bars*, n = 6). SPT was run for 72 h. Results (sucrose intake/total intake ×100) are presented as mean values ±SEM (p>0,05;Student's t-test). Both genotypes display similar behavioral response.

The diminished ability to experience pleasure, called anhedonia, can be behaviorally measured by a decrease in intake of and preference for sweet solutions. Anhedonia is a diagnostic criterion for major depression in the DSM-IVR, and acute NMDAR antagonism reduces anhedonic behavior [44]. Chronic exposure to mild unpredictable stress has been found to reduce the consumption of, and preference for, highly palatable sucrose solution in rodents, a behavior that is reversed by antidepressants [45], [46]. We hypothesized that NMDAR in PV neurons could control the ability to properly value rewarding stimuli. We therefore characterized the response of PV-Cre+/NR1f/f and NR1f/f mice on a standard sucrose preference test, a preclinical measure of anhedonia in rodents [47] (Fig. 6).

We first tested the mice on a water intake test (WIT) aimed to measure the amount (gr) of water consumed in 24 h; subsequently the sucrose preference test (SPT) was running for 72 h (Fig. 6A). We found that both genotypes consumed similar amounts of water: 1.67±0.1 gr NR1f/f (*black bars*) and 1.87±0.1 gr PV-Cre+/NR1f/f (*red bars*) mice (p>0.05 Student's *t*-test) (Fig. 6B).When we performed the SPT we found that PV-Cre+/NR1f/f mice displayed similar preference (*red bars*, 83.8±2.6%) for and consumption of a 2% sucrose solution over a 72 h period as their littermate controls (NR1f/f *black bars*, 77.1±4.2%, p>0.05 Student's *t*-test) (Fig. 6C).

## Discussion

In the present study we have analyzed the role of NMDAR-dependent neurotransmission in GABAergic PV interneurons for the acute antidepressant properties of NMDARs antagonists as well as for the regulation of depression-like behavior. It has been proposed that PV fast-spiking interneurons could play an important role in the antidepressant effects of ketamine [35]. A possible mechanism for the antidepressant properties of NMDAR antagonists is the disinhibition of glutamatergic neurons of the medial prefrontal cortex (mPFC), through a decrease in the activity of PV fast-spiking interneurons [32]. In accordance with this theory, acute ketamine administration rapidly increases glutamatergic neurotransmission in the prefrontal cortex of rodents, a process most likely mediated by presynaptic NMDA autoreceptors and/or NMDAR expressed on GABAergic interneurons [40], which are known to shape circuit function by changing the balance between excitation and inhibition [41].

Although NMDARs are widely localized in the brain, the mPFC has been proposed to be a critical region mediating the antidepressant effects of NMDAR antagonists. However, although local infusion of MK801 into the mPFC is sufficient to increase c-fos protein expression (used as a marker of neuronal activation) in various subcortical regions [42], and systemic administration of MK801 is known to increase the firing of pyramidal neurons of the mPFC of awake rats [42], the direct infusion of ketamine into the mPFC is not sufficient to produce anti-depressant response in rats [43].

In this study we took advantage of genetically modified PV-Cre+/NR1f/f mice that lack NMDARs specifically in PV cells to directly probe the connection between NMDAR-dependent function of PV neurons and depression-like behavior.

Specifically, in this study we aimed a) to address the role of the NMDARs on PV cells on the spontaneous behavior of mice subjected to two different paradigms: the despair-based forced swimming test (FST) and the reward based- sucrose preference test (SPT) and b) to determine whether the NMDAR-dependent activity of PV cells is required for the fast-acting antidepressant action of NMDAR antagonists such as ketamine and MK801 in the FST.

We established a repeated exposure to a FST paradigm where repeated testing of mice does not in itself result in increased depressive behavior, but animals display stable baseline behavioral responses. This paradigm therefore allows for evaluation of the acute as well as the sustained antidepressant effects of a single exposure to drugs or interventions in a group of animals. Our results show that immobility and swimming time scored in the FST at all time points evaluated was similar between mice lacking the NMDAR specifically in PV neurons and their respective littermates controls. Importantly, we also found that both ketamine and MK801, at doses producing antidepressant-like activity, had similar behavioral effects in our mutant mice.

Reduction in sucrose consumption in a sucrose preference test has been used as an indicator of anhedonia that is associated with depression-like behavior. Acute blockage of NMDA receptors by MK801 produces a deficit in sucrose preference in rats [44]. We therefore studied the preference for a sucrose solution in mice lacking the NMDAR in PV neurons compared to control mice and did not observe any difference in sucrose preference.

In summary, our results suggest that the NMDAR-dependent activity of PV neurons is not the primary target for the rapid and sustained antidepressant effect of NMDAR antagonists, and that NMDAR-dependent activity of PV neurons does not regulate mood-related behaviors. We cannot exclude that GABAergic PV interneurons are relevant to depression through NMDAR-independent pathways or by regulating the emotional state in other mood-related disorders

The GABAergic system displays great complexity in terms of neuronal subtypes and receptor expression, and controls a number of cognitive and emotional processes in different brain regions [48]. Based on findings in human post-mortem brain material from depressed patients [18]–[20], it is possible that other types of GABAergic neurons could be of relevance for major depression and for the antidepressant action of ketamine. It will be of interest to characterize the contribution of the somatostatin-expressing interneurons as they control excitability of cortical circuits and information processing [49]. Therefore, further studies are needed to clarify the role of subtypes of GABAergic interneurons for the antidepressant effect of NMDAR antagonists.
