# Correction: Mice Lacking NMDA Receptors in Parvalbumin Neurons Display Normal Depression-Related Behavior and Response to Antidepressant Action of NMDAR Antagonists

**DOI:** 10.1371/journal.pone.0091486

**Published:** 2014-02-28

**Authors:** The PLOS ONE Staff

The name of the second author is incorrectly represented in the Citation. The correct Citation is: Pozzi L, Pollak Dorocic I, Wang X, Carlén M, Meletis K (2014) Mice Lacking NMDA Receptors in Parvalbumin Neurons Display Normal Depression-Related Behavior and Response to Antidepressant Action of NMDAR Antagonists. PLoS ONE 9(1): e83879. doi:10.1371/journal.pone.0083879
